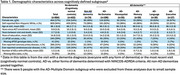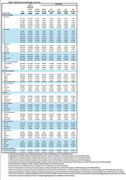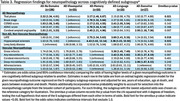# Cognitively defined AD dementia subgroups have different neuropathology findings in a community‐based autopsy cohort

**DOI:** 10.1002/alz70857_106090

**Published:** 2025-12-25

**Authors:** Caitlin S Latimer, Rod L Walker, Marika Bogdani, C. Dirk Keene, Shubhabrata Mukherjee, Seo‐Eun Choi, Michael L. Lee, Connie Nakano, Kelly Ehrlich, Linda K. McEvoy, Paul K Crane

**Affiliations:** ^1^ University of Washington, Seattle, WA, USA; ^2^ Kaiser Permanente Washington Health Research Institute, Seattle, WA, USA; ^3^ Department of Laboratory Medicine and Pathology, University of Washington, Seattle, WA, USA; ^4^ Department of Medicine, University of Washington, Seattle, WA, USA; ^5^ Department of Medicine, University of Washington School of Medicine, Seattle, WA, USA; ^6^ Department of General Internal Medicine, University of Washington School of Medicine, Seattle, WA, USA

## Abstract

**Background:**

Alzheimer's disease (AD) dementia may be heterogenous. Subgrouping can consider relative performance on cognitive tests at AD dementia diagnosis; we compared neuropathology findings across subgroups.

**Method:**

Adult Changes in Thought (ACT) is a prospective cohort study of older adults. Dementia free individuals ^3^age 65 are evaluated every two years with the Cognitive Abilities Screening Instrument (CASI). Those with low scores receive a clinical evaluation with an extensive neurocognitive battery; all data are reviewed at a multidisciplinary consensus conference. CASI and neurocognitive battery data are processed for composite memory, executive functioning, language, and visuospatial scores, which are used for determining AD dementia sub‐groups among participants diagnosed with probable or possible AD per NINCDS‐ADRDA criteria. At death, consenting participants receive a standardized postmortem evaluation. For these analyses we considered guideline‐based assessments of Alzheimer's type pathology (CERAD score, Braak stage, Thal phase, and ADNC score) and cerebral amyloid angiopathy, LATE, Lewy bodies, hippocampal sclerosis, atherosclerosis, arteriolosclerosis, deep and cerebral microinfarcts, and gross infarcts. For each neuropathology outcome we used an ordinal logistic regression model to estimate odds of higher levels of neuropathology associated with each subgroup, adjusted for sex, education, age at death, and time between diagnosis and death. Models additionally incorporated inverse probability weights to account for selection into the neuropathology sample from the broader cohort of participants.

**Result:**

There were 864 people with autopsy data, 432 were cognitively normal, 74 had some other form of dementia, and 358 had AD dementia, with 196 AD‐No Domain, 55 AD‐Visuospatial, 50 AD‐Memory, 30 AD‐Language, and 27 AD‐Executive. Demographic and clinical characteristics are in Table 1. Frequency of neuropathology findings for each subgroup are shown in Table 2. Regression results for the subgroups are shown in Table 3. Thal phases were highest in AD‐Memory and lowest in AD‐Visuospatial. LATE stage and hippocampal sclerosis were highest in AD‐Language and AD‐Memory and lowest in AD‐Visuospatial.

**Conclusion:**

We previously showed atrophy at AD diagnosis differed across subgroups. Here, patterns of neuropathology also appear to differ across subgroups. Further research is warranted on whether other natural history characteristics differ across cognitively defined subgroups.